# High fibrinogen-to-albumin ratio with type 2 diabetes mellitus is associated with poor prognosis in patients undergoing percutaneous coronary intervention: 5-year findings from a large cohort

**DOI:** 10.1186/s12933-022-01477-w

**Published:** 2022-03-21

**Authors:** Peizhi Wang, Deshan Yuan, Ce Zhang, Pei Zhu, Sida Jia, Ying Song, Xiaofang Tang, Jingjing Xu, Tianyu Li, Guyu Zeng, Xueyan Zhao, Yuejin Yang, Bo Xu, Runlin Gao, Jinqing Yuan

**Affiliations:** 1 Department of Cardiology, Center for Coronary Heart Disease, Fuwai Hospital, National Center for Cardiovascular Diseases, Chinese Academy of Medical Sciences and Peking Union Medical College, No. 167, Beilishi Road, Xicheng District, Beijing, 100037 China; 2 Catheterization Laboratories, Fuwai Hospital, National Center for Cardiovascular Diseases, Chinese Academy of Medical Sciences and Peking Union Medical College, Beijing, China; 3 National Clinical Research Center for Cardiovascular Diseases, Fuwai Hospital, National Center for Cardiovascular Diseases, Chinese Academy of Medical Sciences and Peking Union Medical College, Beijing, China

**Keywords:** Fibrinogen‑to‑albumin ratio, Type 2 diabetes mellitus, Prognosis, Percutaneous coronary intervention

## Abstract

**Background:**

Inflammation plays a crucial role in coronary atherosclerosis progression, and growing evidence has demonstrated that the fibrinogen-to-albumin ratio (FAR), as a novel inflammation biomarker, is associated with the severity of coronary artery disease (CAD). However, the long-term risk of cardiovascular events remains indistinct in patients with different level of FAR and different glycemic metabolism status. This study was to assess 5-year clinical outcomes of diabetic and non-diabetic patients who underwent percutaneous coronary intervention (PCI) with different level of FAR.

**Methods:**

We consecutively enrolled 10,724 patients with CAD hospitalized for PCI and followed up for the major adverse cardiac and cerebrovascular events (MACCE) covering all-cause mortality, cardiac mortality, non-fatal myocardial infarction, non-fatal ischemic stroke, and unplanned coronary revascularization. FAR was computed using the following formula: Fibrinogen (g/L)/Albumin (g/L). According to the optimal cut-off value of FAR for MACCE prediction, patients were divided into higher level of FAR (FAR-H) and lower level of FAR (FAR-L) subgroups, and were further categorized into four groups as FAR-H with DM and non-DM, and FAR-L with DM and non-DM.

**Results:**

5298 patients (58.36 ± 10.36 years, 77.7% male) were ultimately enrolled in the present study. A total of 1099 (20.7%) MACCEs were documented during the 5-year follow-up. The optimal cut-off value of FAR was 0.0783 by the surv_cutpoint function. Compared to ones with FAR-H and DM, patients with FAR-L and non-DM, FAR-H and non-DM, FAR-L and DM had decreased risk of MACCEs [adjusted hazard ratio (HR): 0.75, 95% confidence interval (CI) 0.64–0.89, P = 0.001; HR: 0.78, 95% CI 0.66–0.93, P = 0.006; HR: 0.81, 95% CI 0.68–0.97, P = 0.019; respectively]. Notably, non-diabetic patients with lower level of FAR also had lower all-cause mortality and cardiac mortality risk than those in the FAR-H/DM group (HR: 0.41, 95% CI 0.27–0.63, P < 0.001; HR: 0.30, 95% CI 0.17–0.53, P < 0.001; respectively). Multivariate Cox proportional hazards regression analysis also indicated the highest risk of MACCEs in patients with FAR-H and DM than others (P for trend = 0.005). In addition, post-hoc analysis revealed consistent effects on 5-year MACCE across various subgroups.

**Conclusion:**

In this real-world cohort study, higher level of FAR combined with DM was associated with worse 5-year outcomes among patients with CAD undergoing PCI. The level of FAR may help to identify high-risk individuals in this specific population, where more precise risk assessment should be performed.

**Supplementary Information:**

The online version contains supplementary material available at 10.1186/s12933-022-01477-w.

## Introduction

Inflammation plays a crucial role in coronary atherosclerosis progression [1]. Increasing evidence has indicated that higher levels of inflammatory biomarkers are associated with increased adverse cardiovascular events in patients with coronary artery disease (CAD) [2–5]. Previous studies suggested that fibrinogen (FIB), a biomarker of inflammation as well as a core component in the coagulation pathway, was an independent risk factor and might predict cardiovascular events in patients with CAD [3, 6]. Besides, our prior findings also indicated that higher level of FIB was strongly related to increased risk of long-term all-cause and cardiac mortality among CAD patients undergoing percutaneous coronary intervention (PCI), especially in those with diabetes mellitus (DM) [7]. As the most abundant plasma protein, albumin is a negative acute‑phase reactant produced in the liver, whose serum concentration is associated with inflammatory and hemostatic processes [8, 9]. Moreover, serum albumin level was inversely associated with cardiovascular mortality and hypoalbuminemia might predict the no-flow phenomenon in patients with acute myocardial infarction (AMI) after PCI [10, 11]. Therefore, both fibrinogen and albumin are important equivalent of hemorheological and inflammatory alterations. Recently, several publications confirmed that fibrinogen-to-albumin ratio (FAR), which comprised these two indicators above, was a well-established prognostic factor in esophageal, liver and breast cancers [12–14], and had a closely association with the severity of coronary lesions as well as short-term prognosis in patients with CAD [15–17].

Inflammation is regarded as the common antecedent of atherosclerosis and diabetes [2, 18]. Type 2 diabetes mellitus, a well-established risk factor of CAD, has been previously demonstrated to be closely associated with greater atherosclerotic plaque burden and increased risk of adverse cardiovascular events [19–21]. However, to date, insufficient literature has investigated the relationship between the level of FAR, glucose metabolism and long-term prognosis in the CAD population after PCI. In the light of the above, the present study was conducted to evaluate the relationship between FAR and glycemic metabolism indices, and further determine the joint effect of FAR and DM on long-term major adverse cardiac and cerebrovascular events (MACCE) in CAD patients undergoing PCI.

## Methods

### Study design

**Fig. 1:**
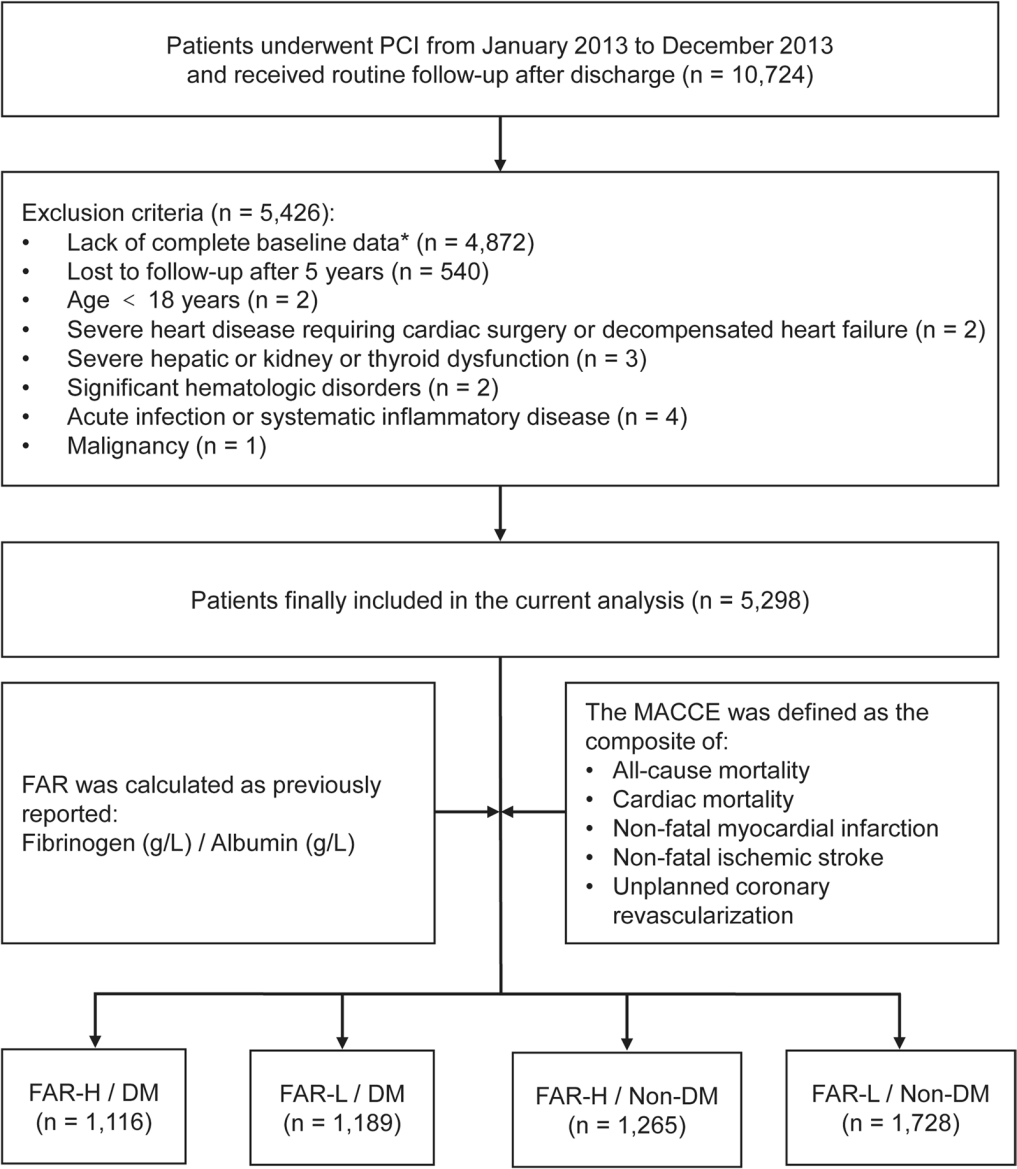
Study flowchart. *PCI* percutaneous coronary intervention*, MACCE* major adverse cardiac and cerebrovascular events*, FAR* fibrinogen-to-albumin ratio*, DM* diabetes mellitus * 4607 patients with missing fibrinogen values and 265 patients with missing fasting blood glucose or HbA1c levels were excluded

From January 2013 to December 2013, a total of 10,724 consecutive CAD patients were hospitalized for PCI in Fuwai Hospital, National Center for Cardiovascular Diseases, Chinese Academy of Medical Sciences, Beijing, China. Patients with missing baseline and follow-up data, age < 18 years and/or other exclusion criteria were excluded (detailed recruitment process shown in Fig. 1). A comparison of the baseline characteristics and crude outcomes between the non-participants and participants was presented in Additional file 1: Table S1. Overall, 5298 patients were ultimately included in the present analysis and assigned to the FAR-H/DM (n = 1116), FAR-L/DM (n = 1189), FAR-H/Non-DM (n = 1265) and FAR-L/Non-DM (n = 1728) groups, due to the optimal cut-off value of FAR and different glycemic metabolism status.

Enrolled patients were routinely followed up through telephone interview or examination of medical records at five time points (1-month, 6-month, 1-year, 2-year, and 5-year after discharge) by well-trained research coordinators, who were blinded to the objectives of the current study. The primary endpoint was defined as a composite of the MACCE covering all-cause mortality, cardiac mortality, non-fatal myocardial infarction, non-fatal ischemic stroke and unplanned coronary revascularization [22]. The secondary outcome was each component of MACCE. All events were adjudicated centrally by 2 independent and experienced cardiologists, who were unaware of the study protocol. Conflicts were resolved by turning to a third experienced cardiologist.

This study complied with the Declaration of Helsinki and was endorsed by the Ethics Committee of The Fuwai Hospital, National Center for Cardiovascular Diseases, Beijing, China. The informed written consent was obtained from all patients before the intervention.

### Treatment and procedure

PCI was performed by interventional cardiologists who were blind to the study protocol, in line with current practice guidelines in China. The choice of equipment and detailed strategies during coronary intervention was at the discretion of the operating physicians. Before the procedure, elective PCI patients received oral administration of aspirin (300 mg) and ticagrelor (loading dose 180 mg) or clopidogrel (loading dose 300 mg) at least 24 h, unless on long-term P2Y12 inhibitor treatment. Patients presenting as acute coronary syndrome (ACS) scheduled for PCI received the same dose of aspirin and ticagrelor (loading dose 180 mg) or clopidogrel (loading dose 300–600 mg) as soon as possible. Unfractionated heparin (100 U/kg) was administered before PCI, however, the use of glycoprotein IIb/IIIa inhibitors was at the cardiologist’s judgment during the procedure. After the catheterization, the dual antiplatelet therapy including aspirin (100 mg daily) and ticagrelor (90 mg, twice daily) or clopidogrel (75 mg, daily) were recommended for at least 12 months.

### Definitions

Diabetes mellitus was diagnosed by fasting blood glucose (FBG) ≥ 7.0 mmol/L (126 mg/dL), or hemoglobin A1c (HbA1c) levels ≥ 6.5%, or 2-h blood glucose of oral glucose tolerance test ≥ 11.1 mmol/L (200 mg/dL), or previous definite diagnosis of DM with hypoglycemic drugs treatment [23]. FAR was defined as the ratio of the preoperative plasma fibrinogen concentration (g/L) to the preoperative plasma albumin level (g/L). Hypertension (HT) was defined as newly confirmation more than twice on different days by systolic blood pressure ≥ 140 mmHg and/or diastolic blood pressure ≥ 90 mmHg during the baseline hospitalization, or known HT with antihypertensive therapy [24]. Fasting total cholesterol (TC) ≥ 5.2 mmol/L, low-density lipoprotein cholesterol (LDL-C) ≥ 3.4 mmol/L, high-density lipoprotein cholesterol (HDL-C) < 1.0 mmol/L, triglyceride (TG) ≥ 1.7 mmol/L, and/or long-term treatment with lipid-lowering drugs were considered criteria for dyslipidemia [25]. Body mass index (BMI) was calculated as weight (kg)/[height (m)]^2^. Previous medical history of MI, PCI, stroke, and peripheral artery disease (PAD), smoking history, family history of CAD, and medications at admission were collected from self-reported information and then confirmed by relevant medical records.

Coronary procedural information was interpreted and recorded by two independent experienced operating physicians and disagreement was resolved by consensus. Based on the coronary angiography, left main disease was defined as stenosis of ≥ 50% in left main coronary artery and three-vessel disease was diagnosed by stenosis of ≥ 50% in all three main coronary arteries (left anterior coronary artery, left circumflex artery and right coronary artery). Chronic total occlusion (CTO) was defined as complete obstruction of a native coronary artery for more than 3 months with thrombolysis in myocardial infarction (TIMI) flow grade 0 [26]. The synergy between PCI with taxus and cardiac surgery (SYNTAX) score was calculated using an online calculator (http://www.syntaxscore.com/) to evaluate the coronary lesion complexity by a dedicated research group blinded to the clinical data. Complete revascularization was considered successful if residual stenosis < 30% with TIMI flow grade 3 at the end of the PCI procedure was obtained according to visual estimation of the angiograms [26].

### Laboratory tests measurements

Fasting blood samples were drawn from each patient within 24 h after admission and all of them were stored in −80 °C refrigerators until test. Stago autoanalyzer with the STA fibrinogen kit (Diagnostica Stago, Taverny, France) was used to measure the concentrations of plasma fibrinogen. Serum albumin was measured using an automated chemistry analyzer (AU5400, Olymus, Japan) by the bromocresol green dye method. Tosoh Automated Glycohemoglobin Analyzer (HLC-723G8, Tokyo, Japan) was used to measure the HbA1c levels. The concentrations of fast blood glucose were measured by the enzymatic hexokinase method. Other laboratory indices, including lipid profiles (TG, TC, HDL-C and LDL-C), estimated glomerular filtration rate (eGFR), creatinine, high-sensitivity C-reactive protein (hs-CRP) were examined with standard biochemical techniques at the core laboratory in Fuwai Hospital. According to modified Simpson’s rule, left ventricular ejection fraction (LVEF) was measured from two-dimensional echocardiography.

### Statistical analyses

Continuous variates were described as mean with standard deviation and nominal variates were summarized as frequency with percentage. Comparison of continuous and categorical variates among different groups was analyzed by Student’s t-test or Analysis of variance and Chi-square test or Fisher’s exact test, as appropriate. The surv_cutpoint function was used to determine the optimal cut-off value for FAR. Pearson correlation and linear regression analysis were constructed to evaluate the correlation between FAR and glycemic metabolism indices (FBG and HbA1c). The risks of MACCE in different subgroups were presented by Kaplan–Meier survival curves and compared by log-rank test. The associations among FAR, DM, and 5-year clinical outcomes were analyzed by unadjusted and adjusted Cox regression analyses. Hazard ratios (HRs) and 95% confidence interval (CI) were reported. In the multivariate Cox analysis, age, sex, BMI, hypertension, previous MI, previous PCI, previous stroke, eGFR, LVEF, Left main (LM)/three-vessel disease, and SYNTAX score were adjusted because of their statistical significance (P < 0.05) in univariate analysis or clinical importance (Additional file 1: Table S2). Furthermore, exploratory analyses were performed to assess the effect of FAR and glycemic metabolism status in different subgroups on the primary endpoint in specific subsets and shown as the forest plot. In addition, the continuous relationship between FAR and the risk of MACCE was illustrated by restricted cubic spline and examined by the likelihood ratio test. Statistical analyses were performed using SPSS software (version 26.0; IBM Corp., Armonk, N.Y., United States), RStudio software (version 2021.09.0; http://www.rstudio.org/), and GraphPad Prism software (version 8.0.1; GraphPad Software, Inc., La Jolla, CA, United States). A two-tailed P value < 0.05 was considered statistical significance.

## Results

Overall, 5298 patients (58.36 ± 10.36 years, 77.7% male) who met the enrollment criteria and completed the 5-year follow-up were ultimately enrolled in the present study. During the 5-year follow-up (interquartile range: 5.0–5.1 years), 206 (3.89%) all-cause mortality, 310 (5.85%) non-fatal MI, 184 (3.47%) non-fatal ischemic stroke, 671 (12.67%) unplanned coronary revascularization and 1099 (20.7%) MACCEs were recorded.

### Baseline characteristics

The baseline characteristics of enrolled patients and groups stratified by the occurrence of MACCEs were summarized in Table 1. Patients with each component of the primary endpoint presented older and higher level of FAR, FIB, FBG, HbA1c, hs-CRP, creatinine, eGFR and LVEF. In addition, they also had higher prevalence of DM, HT, previous MI, previous PCI, previous stroke, nitrate at admission and insulin before hospitalization. As for the angiographic findings, patients in the MACCEs subset were more likely to have LM/three-vessel disease, and target lesions in RCA and LCX. Moreover, the SYNTAX score was significantly higher in participants with adverse prognosis.

**Table 1:** Baseline demographics and angiographic characteristics of entire population stratified by the primary endpoint

Variable	Total population (n = 5298)	Non-MACCEs (n = 4199)	MACCEs (n = 1099)	P value
FAR	0.081 ± 0.022	0.080 ± 0.022	0.082 ± 0.024	0.010
Four subgroups, n (%)				< 0.001
FAR-H/DM	1116 (21.1)	829 (19.7)	287 (26.1)	
FAR-L/DM	1189 (22.4)	941 (22.4)	248 (22.6)	
FAR-H/Non-DM	1265 (23.9)	1018 (24.2)	247 (22.5)	
FAR-L/Non-DM	1728 (32.6)	1411 (33.6)	317 (28.8)	
Baseline characteristics				
Age, years	58.36 ± 10.36	58.12 ± 10.26	59.30 ± 10.68	0.001
Male, n (%)	4,119 (77.7)	3,246 (77.3)	873 (79.4)	0.130
BMI, kg/m^2^	25.89 ± 3.15	25.92 ± 3.15	25.81 ± 3.15	0.338
DM, n (%)	2305 (43.5)	1770 (42.2)	535 (48.7)	< 0.001
Hypertension, n (%)	3386 (63.9)	2629 (62.6)	757 (68.9)	< 0.001
Dyslipidemia, n (%)	3657 (69.0)	2889 (68.8)	768 (69.9)	0.491
Smoking history, n (%)	3107 (58.6)	2435 (58.0)	672 (61.1)	0.059
Family history of CAD, n (%)	1222 (23.1)	948 (22.6)	274 (24.9)	0.101
Previous MI, n (%)	1036 (19.6)	791 (18.8)	245 (22.3)	0.010
Previous PCI, n (%)	1229 (23.2)	914 (21.8)	315 (28.7)	< 0.001
Previous stroke, n (%)	556 (10.5)	419 (10.0)	137 (12.5)	0.017
Previous PAD, n (%)	150 (2.8)	120 (2.9)	30 (2.7)	0.820
Clinical presentation, n (%)				0.435
CCS	2258 (42.6)	1801 (42.9)	457 (41.6)	
ACS	3040 (57.4)	2398 (67.1)	642 (58.4)	
Laboratory tests				
FIB, g/L	3.38 ± 0.83	3.37 ± 0.82	3.43 ± 0.85	0.047
Albumin, g/L	42.40 ± 3.83	42.45 ± 3.84	42.22 ± 3.79	0.079
FBG, mmol/L	6.02 ± 1.97	5.99 ± 1.93	6.16 ± 2.11	0.012
HbA1c, %	6.59 ± 1.21	6.56 ± 1.21	6.72 ± 1.21	< 0.001
TG, mmol/L	1.80 ± 1.09	1.80 ± 1.10	1.80 ± 1.02	0.895
TC, mmol/L	4.17 ± 1.09	4.17 ± 1.08	4.16 ± 1.90	0.883
HDL-C, mmol/L	1.02 ± 0.27	1.02 ± 0.28	1.01 ± 0.27	0.158
LDL-C, mmol/L	2.48 ± 0.91	2.48 ± 0.91	2.48 ± 0.91	0.971
hs-CRP, mg/L	3.07 ± 3.68	2.99 ± 3.63	3.36 ± 3.87	0.006
Creatinine, μmol/L	75.33 ± 15.95	74.98 ± 15.50	76.68 ± 17.52	0.004
eGFR, mL/min/1.73 m^2^	91.63 ± 15.10	92.01 ± 14.72	90.18 ± 16.41	0.001
LVEF, %	63.23 ± 7.04	63.38 ± 6.92	62.67 ± 7.47	0.005
Medications at admission				
Aspirin, n (%)	5245 (99.0)	4157 (99.0)	1088 (99.0)	0.998
Clopidogrel, n (%)	5286 (99.8)	4190 (99.8)	1096 (99.7)	0.716
β-blocker, n (%)	4829 (91.1)	3822 (91.0)	1007 (91.6)	0.528
CCB, n (%)	2485 (46.9)	1941 (46.2)	544 (49.5)	0.053
Statins, n (%)	5112 (96.5)	4054 (96.5)	1058 (96.3)	0.656
Nitrate, n (%)	5162 (97.4)	4082 (97.2)	1080 (98.3)	0.048
Insulin, n (%)	613 (11.6)	456 (10.9)	157 (14.3)	0.002
Coronary procedural information				
LM/three-vessel disease, n (%)	2382 (45.0)	1806 (43.0)	576 (52.4)	< 0.001
Chronic total occlusion, n (%)	377 (7.1)	291 (6.9)	86 (7.8)	0.304
Target vessel territory, n (%)				< 0.001
LAD	2512 (47.4)	2073 (49.4)	439 (39.9)	
LCX	955 (18.0)	739 (17.6)	216 (19.7)	
RCA	1757 (33.2)	1334 (31.8)	423 (38.5)	
Number of stents	1.77 ± 0.89	1.76 ± 0.90	1.79 ± 0.86	0.303
SYNTAX score	11.79 ± 9.06	11.61 ± 7.90	12.47 ± 8.62	0.003
Complete revascularization, n (%)	5268 (99.4)	4179 (99.5)	1089 (99.1)	0.088
DES implantation, n (%)	5226 (98.6)	4148 (98.8)	1078 (98.1)	0.076

*MACCE* major adverse cardiac and cerebrovascular events, *FAR* fibrinogen-to-albumin ratio, *BMI* body mass index, *DM* diabetes mellitus, *CAD* coronary artery disease, *MI* myocardial infarction, PCI percutaneous coronary intervention, PAD peripheral artery disease, *CCS* chronic coronary syndrome, *ACS* acute coronary syndrome, *FIB* fibrinogen, *FBG* fasting blood glucose, *HbA1c* glycosylated hemoglobin A1c, *TG* triglyceride, *TC* total cholesterol, *HDL-C* high-density lipoprotein cholesterol, *LDL-C* low-density lipoprotein cholesterol, *hs-CRP* high-sensitivity C-reactive protein, *eGFR* estimated glomerular filtration rate, *LVEF* left ventricular ejection fraction, *CCB* calcium channel blocker, *LM* left main artery, *LAD* left anterior descending artery, *LCX* left circumflex artery, *RCA* right coronary artery, *SYNTAX* synergy between PCI with taxus and cardiac surgery, *DES* drug-eluting stent

### Comparison of clinical data among four groups

Based on the surv_cutpoint function of the R package survminer in the R programming language, the optimal cut-off point of FAR is 0.0783. Thus, baseline characteristics of four subgroups according to the level of FAR and glycemic metabolism status were shown in Table 2. Compared with patients in the FAR-H/DM group, those in other three subgroups tended to be younger and male, with a lower proportion of comorbidities, such as HT, dyslipidemia, and previous stroke. Laboratory indices including FIB, FBG, HbA1c, TC, LDL-C, hs-CRP, creatine, eGFR and LVEF were significantly higher in patients with higher level of FAR combined with DM, while the level of HDL-C was relatively lower. Meanwhile, patients in other three subsets were less likely to have LM/three-vessel disease, target lesions in RCA and LCX, and higher SYNTAX score, when compared with those in the FAR-H/DM group.

**Table 2:** Baseline demographics and angiographic characteristics stratified by low or high FAR and different glycemic metabolism status

Variable	FAR-H/DM (n = 1116)	FAR-L/DM (n = 1189)	FAR-H/Non-DM (n = 1265)	FAR-L/Non-DM (n = 1728)	P value
FAR	0.099 ± 0.021	0.067 ± 0.009	0.098 ± 0.021	0.066 ± 0.008	< 0.001
Baseline characteristics					
Age, years	60.86 ± 10.03	58.20 ± 9.70	59.54 ± 10.75	56.01 ± 10.21	< 0.001
Male, n (%)	779 (69.8)	957 (80.5)	937 (74.1)	1,146 (83.7)	< 0.001
BMI, kg/m^2^	26.20 ± 3.22	26.37 ± 3.01	25.54 ± 3.09	25.63 ± 3.18	< 0.001
DM, n (%)	1116 (48.4)	1189 (51.6)	–	–	< 0.001
Hypertension, n (%)	784 (70.3)	807 (67.9)	788 (62.3)	1007 (58.3)	< 0.001
Dyslipidemia, n (%)	826 (74.0)	868 (73.0)	818 (64.7)	1145 (66.3)	< 0.001
Smoking history, n (%)	624 (55.9)	697 (58.6)	732 (57.9)	1054 (61.0)	0.053
Family history of CAD, n (%)	230 (20.6)	276 (23.2)	282 (22.3)	434 (25.1)	0.040
Previous MI, n (%)	236 (21.1)	258 (21.7)	198 (15.7)	344 (19.9)	0.001
Previous PCI, n (%)	274 (24.6)	341 (28.7)	220 (17.4)	394 (22.8)	< 0.001
Previous stroke, n (%)	162 (14.5)	133 (11.2)	120 (9.5)	141 (8.2)	< 0.001
Previous PAD, n (%)	41 (3.7)	43 (3.6)	24 (1.9)	42 (2.4)	0.014
Clinical presentation, n (%)					< 0.001
CCS	443 (39.7)	583 (49.0)	438 (34.6)	794 (45.9)	
ACS	673 (60.3)	606 (51.0)	827 (65.4)	934 (54.1)	
Laboratory tests					
FIB, g/L	4.05 ± 0.77	2.91 ± 0.42	3.99 ± 0.78	2.84 ± 0.39	< 0.001
Albumin, g/L	41.22 ± 3.75	43.52 ± 3.57	41.12 ± 3.67	43.34 ± 3.66	< 0.001
FBG, mmol/L	7.28 ± 2.43	7.22 ± 2.40	5.07 ± 0.56	5.08 ± 0.58	< 0.001
HbA1c, %	7.67 ± 1.40	7.36 ± 1.21	5.93 ± 0.31	5.86 ± 0.35	< 0.001
TG, mmol/L	1.85 ± 1.13	1.97 ± 1.40	1.68 ± 0.85	1.72 ± 0.93	< 0.001
TC, mmol/L	4.22 ± 1.13	4.14 ± 1.09	4.19 ± 1.03	4.14 ± 1.09	0.173
HDL-C, mmol/L	0.98 ± 0.26	1.02 ± 0.26	1.01 ± 0.27	1.06 ± 0.29	< 0.001
LDL-C, mmol/L	2.54 ± 0.94	2.41 ± 0.89	2.52 ± 0.88	2.45 ± 0.93	0.002
hs-CRP, mg/L	5.08 ± 4.48	1.71 ± 2.03	4.82 ± 4.38	1.42 ± 1.73	< 0.001
Creatinine, μmol/L	76.44 ± 19.39	74.83 ± 15.27	75.71 ± 16.55	74.70 ± 13.24	0.019
eGFR, mL/min/1.73 m^2^	88.16 ± 17.12	92.39 ± 14.89	90.03 ± 15.15	94.52 ± 13.09	< 0.001
LVEF, %	62.12 ± 7.45	63.22 ± 6.85	62.90 ± 7.50	64.19 ± 6.40	< 0.001
Medications at admission, n (%)					
Aspirin, n (%)	1104 (98.9)	1177 (99.0)	1247 (98.6)	1717 (99.4)	0.199
Clopidogrel, n (%)	1114 (99.8)	1185 (99.7)	1264 (99.9)	1723 (99.7)	0.517
β-blocker, n (%)	1108 (91.2)	1115 (93.8)	1146 (90.6)	1550 (89.7)	0.002
CCB, n (%)	566 (50.7)	583 (49.0)	704 (55.7)	960 (55.6)	< 0.001
Statins, n (%)	1069 (95.8)	1140 (95.9)	1226 (96.9)	1677 (97.0)	0.157
Nitrate, n (%)	1088 (97.5)	1153 (97.0)	1232 (97.4)	1689 (97.7)	0.638
Insulin, n (%)	319 (28.6)	294 (24.7)	–	–	< 0.001
Coronary procedural information					
LM/three-vessel disease, n (%)	604 (54.1)	568 (47.8)	525 (41.5)	685 (39.6)	< 0.001
Chronic total occlusion, n (%)	66 (5.9)	86 (7.2)	98 (7.7)	127 (7.3)	0.338
Target vessel territory, n (%)					0.001
LAD	471 (42.2)	540 (45.4)	623 (49.2)	878 (50.8)	
LCX	232 (20.8)	211 (17.7)	207 (16.4)	305 (17.7)	
RCA	396 (35.5)	426 (35.8)	415 (32.8)	520 (30.1)	
Number of stents	1.79 ± 0.86	1.77 ± 0.86	1.76 ± 0.94	1.75 ± 0.89	0.677
SYNTAX score	12.76 ± 8.47	11.77 ± 8.27	12.14 ± 8.22	10.92 ± 7.41	< 0.001
Complete revascularization, n (%)	1106 (99.1)	1185 (99.7)	1256 (99.3)	1721 (99.6)	0.209
DES implantation, n (%)	1099 (98.5)	1175 (98.8)	1253 (99.1)	1699 (98.3)	0.331

*MACCE* major adverse cardiac and cerebrovascular events, *FAR* fibrinogen-to-albumin ratio, *BMI* body mass index, *DM* diabetes mellitus, *CAD* coronary artery disease, *MI* myocardial infarction, PCI percutaneous coronary intervention, PAD peripheral artery disease, *CCS* chronic coronary syndrome, *ACS* acute coronary syndrome, *FIB* fibrinogen, *FBG* fasting blood glucose, *HbA1c* glycosylated hemoglobin A1c, *TG* triglyceride, *TC* total cholesterol, *HDL-C* high-density lipoprotein cholesterol, *LDL-C* low-density lipoprotein cholesterol, *hs-CRP* high-sensitivity C-reactive protein, *eGFR* estimated glomerular filtration rate, *LVEF* left ventricular ejection fraction, *CCB* calcium channel blocker, *LM* left main artery, *LAD* left anterior descending artery, *LCX* left circumflex artery, *RCA* right coronary artery, *SYNTAX* synergy between PCI with taxus and cardiac surgery, *DES* drug-eluting stent

### Relationship between FAR and FBG or HbA1c

Linear regression analysis was conducted to evaluate the correlation between FAR and glycemic metabolism indices (Additional file 1: Table S3). The results showed that both admission FBG (R^2^ = 0.003, Standard β = 0.061, P < 0.001) and HbA1c (R^2^ = 0.019, Standard β = 0.139, P < 0.001) were positively associated with FAR in the whole cohort. Furthermore, both in the DM subgroup and in the non-DM subgroup, there was also a positive relationship between FAR and HbA1c (R^2^ = 0.025, Standard β = 0.101, P < 0.001; R^2^ = 0.010, Standard β = 0.003, P < 0.001, respectively).

### Predictive value of FAR combined with glycemic metabolism status on MACCE and each component

**Fig. 2:**
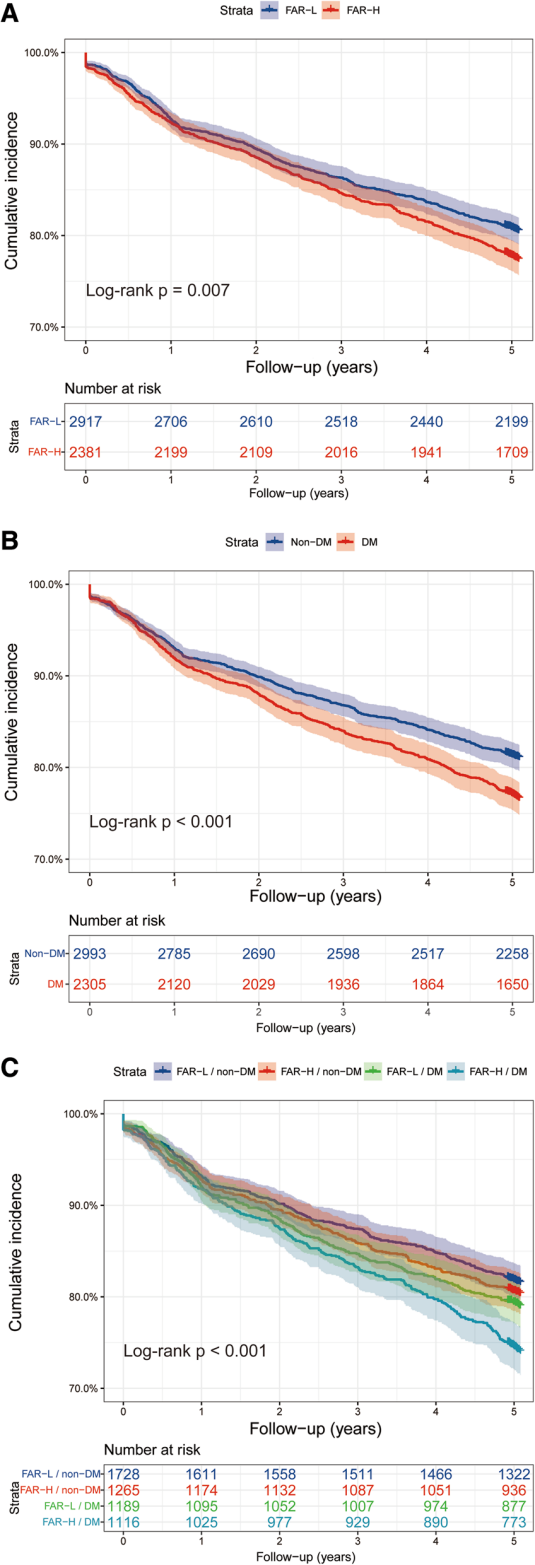
Kaplan–Meier analysis for MACCE according to different FAR levels (**A**), glycemic metabolism status (**B**), and status of both FAR levels and glycemic metabolism (**C**)

**Fig. 3:**
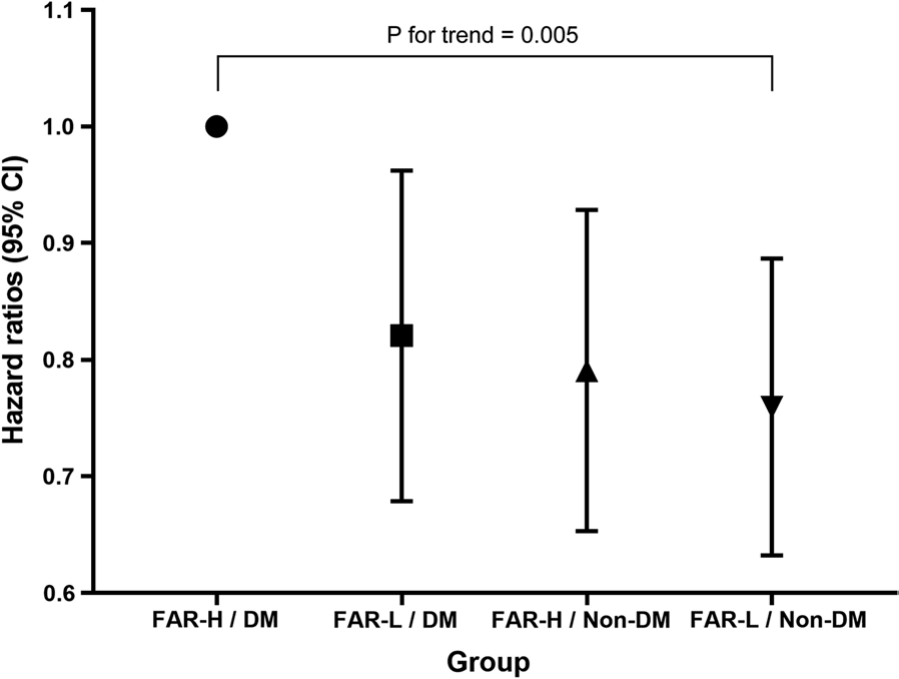
Hazard ratios (95% CIs) for MACCE according to four groups after adjusting for age, sex, BMI, hypertension, previous MI, previous PCI, previous stroke, eGFR, LVEF, LM/three-vessel disease, and SYNTAX score

The incidence of the primary endpoint in FAR-H with DM or non-DM and FAR-L with DM or non-DM group was 25.7% (287/1116), 20.9% (248/1189), 19.5% (247/1265) and 18.3% (317/1728), respectively. As shown in Fig. 2, the Kaplan–Meier analysis curves revealed the highest risk of MACCEs in patients with FAR-H and DM compared with other groups (log-rank test P < 0.001). Furthermore, we assessed the prognostic utility in enrolled patients presented different level of FAR with or without DM by Cox regression analysis (Table 3). In the unadjusted model, patients with FAR-L and DM, FAR-H and non-DM, and FAR-L and non-DM showed decreased risk of MACCEs (HR: 0.80, 95% CI 0.67–0.95, P = 0.009; HR: 0.74; 95% CI 0.63–0.88, P = 0.001; HR: 0.69, 95% CI 0.59–0.81, P < 0.001; respectively) compared to those in FAR-H and DM group (as reference). The results remained statistical significance in multivariate analysis after adjusted for age, sex, BMI, hypertension, previous MI, previous PCI, previous stroke, eGFR, LVEF, LM/three-vessel disease, and SYNTAX score (Table 3; adjusted HR: 0.81, 95% CI 0.68–0.97, P = 0.019; adjusted HR: 0.78, 95% CI 0.66–0.93, P = 0.006; adjusted HR: 0.75, 95% CI 0.64–0.89, P = 0.001; respectively). Particularly, there was a significant risk of the all-cause mortality and the cardiac mortality in patients with FAR-H/DM and FAR-L/Non-DM (Table 3; adjusted HR: 0.75, 95% CI 0.64–0.89, P < 0.001; adjusted HR: 0.30, 95% CI 0.17–0.53, P < 0.001; respectively). Multivariate Cox proportional hazards regression analysis also indicated the highest risk of MACCEs in patients with FAR-H and DM than others (Fig. 3; P for trend = 0.005). Moreover, restricted cubic spline analysis elucidated that there was a linear association between FAR and the risk of MACCE, regardless of the unadjusted and adjusted model (Additional file 1: Fig. S1; all P for non-linear association > 0.05).

**Table 3:** Predictive value of the FAR level and different glycemic metabolism status for primary endpoint and each component in univariate and multivariate analysis

Variables	Events/subjects	Univariate analysis		Multivariate analysis	
		HR (95% CI)	P value	HR (95% CI)	P value
MACCE	1099/5298				
FAR-H/DM	287/1116	Reference	–	Reference	–
FAR-L/DM	248/1189	0.80 (0.67–0.95)	0.009	0.81 (0.68–0.97)	0.019
FAR-H/Non-DM	247/1265	0.74 (0.63–0.88)	0.001	0.78 (0.66–0.93)	0.006
FAR-L/Non-DM	317/1728	0.69 (0.59–0.81)	< 0.001	0.75 (0.64–0.89)	0.001
All-cause mortality	206/5298				
FAR-H/DM	71/1116	Reference	–	Reference	–
FAR-L/DM	44/1189	0.57 (0.39–0.84)	0.004	0.68 (0.47–1.00)	0.053
FAR-H/Non-DM	56/1265	0.69 (0.49–0.98)	0.040	0.72 (0.50–1.03)	0.076
FAR-L/Non-DM	35/1728	0.31 (0.21–0.47)	< 0.001	0.41 (0.27–0.63)	< 0.001
Cardiac mortality	125/5298				
FAR-H/DM	49/1116	Reference	–	Reference	–
FAR-L/DM	27/1189	0.51 (0.32–0.82)	0.005	0.62 (0.39–1.00)	0.052
FAR-H/Non-DM	35/1265	0.63 (0.41–0.97)	0.035	0.67 (0.43–1.05)	0.077
FAR-L/Non-DM	17/1728	0.22 (0.13–0.38)	< 0.001	0.30 (0.17–0.53)	< 0.001
Non-fatal MI	310/5298				
FAR-H/DM	77/1116	Reference	–	Reference	–
FAR-L/DM	67/1189	0.82 (0.61–1.10)	0.187	0.81 (0.58–1.13)	0.221
FAR-H/Non-DM	66/1265	0.75 (0.54–1.04)	0.084	0.80 (0.57–1.12)	0.185
FAR-L/Non-DM	100/1728	0.80 (0.58–1.11)	0.188	0.87 (0.64–1.19)	0.376
Non-fatal ischemic stroke	184/5298				
FAR-H/DM	48/1116	Reference	–	Reference	–
FAR-L/DM	44/1189	0.85 (0.57–1.28)	0.447	0.92 (0.60–1.40)	0.702
FAR-H/Non-DM	47/1265	0.86 (0.57–1.28)	0.455	0.89 (0.59–1.35)	0.593
FAR-L/Non-DM	45/1728	0.59 (0.39–0.89)	0.011	0.70 (0.46–1.08)	0.105
Unplanned coronary revascularization	671/5298				
FAR-H/DM	160/1116	Reference	–	Reference	–
FAR-L/DM	155/1189	0.90 (0.72–1.12)	0.333	0.85 (0.68–1.07)	0.165
FAR-H/Non-DM	142/1265	0.77 (0.62–0.97)	0.024	0.79 (0.63–1.00)	0.050
FAR-L/Non-DM	214/1728	0.84 (0.69–1.04)	0.103	0.82 (0.66–1.02)	0.070

Model adjusted for age, sex, BMI, hypertension, previous MI, previous PCI, previous stroke, eGFR, LVEF, LM/three-vessel disease, and SYNTAX score

*MACCE* major adverse cardiac and cerebrovascular events, *FAR* fibrinogen-to-albumin ratio, *CI* confidence interval, *DM* diabetes mellitus, *MI* myocardial infarction

### Subgroup analysis

**Fig. 4:**
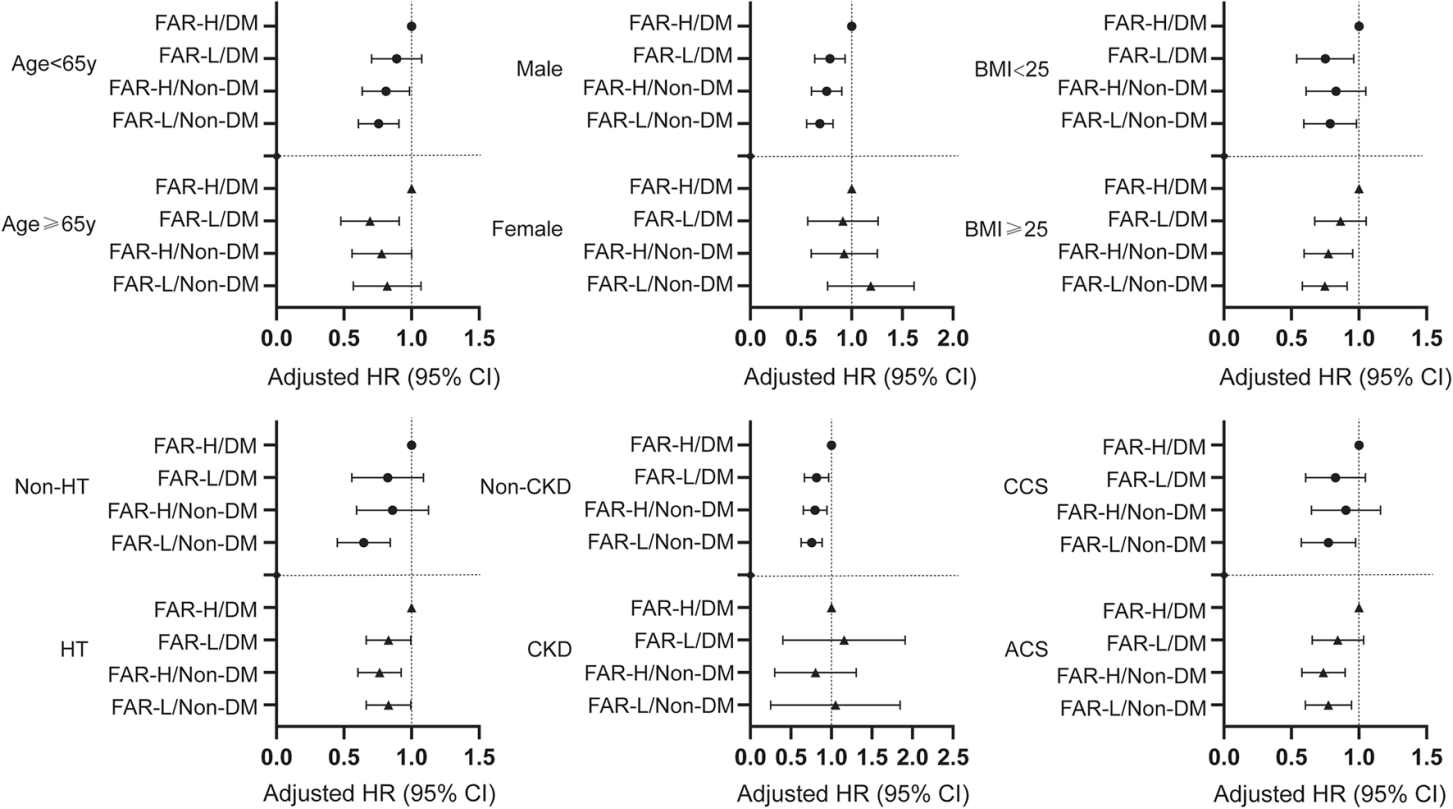
Forest plot of MACCE according to different subgroups. Adjusted model included age, sex, BMI, hypertension, previous MI, previous PCI, previous stroke, eGFR, LVEF, LM/three-vessel disease, and SYNTAX score

Further evaluation of post-hoc subgroup analysis presented comparable interactions following MACCEs between four subsets and those covariates (age, sex, BMI, hypertension, chronic kidney disease, and clinical presentation) (Fig. 4 & Additional file 1: Table S5; all P for interaction > 0.05). Interestingly, patients with FAR-L/DM, FAR-H/non-DM and FAR-L/non-DM showed significantly consistent characteristics in certain subsets (male and non-CKD), when compared to ones in the FAR-H/DM group.

## Discussion

In this real-world, perspective, observational study on a large-scale cohort from China with 5-year follow-up, we examined the prognostic association of type 2 diabetes mellitus with adverse outcomes in patients with different level of the fibrinogen-to-albumin ratio. Our data demonstrated that diabetic patients with higher level of FAR were associated with significantly increased risks of long-term MACCEs compared to ones in other three groups. Furthermore, non-diabetic patients with lower level of FAR also had lower all-cause mortality and cardiac mortality risk than those in the FAR-H/DM group. Multivariate Cox analysis also revealed the highest risk of MACCEs in patients with FAR-H and DM than others. Additionally, compared with patients in the FAR-H/DM group, patients in other three groups showed significantly consistency in certain subsets (male and non-CKD), which may conduct clinical trials for certain therapies. Our findings suggested that more precise risk assessment should be performed in diabetic patients with higher plane of FAR.

FIB, a serum glycoprotein synthesized by the liver with a dimeric molecular structure, plays a crucial role in the inflammatory and coagulation cascade, which contribute to the pathogenesis of coronary atherosclerosis [2, 3]. Many literatures have reported the association between FIB and inflammation involved in progression of CAD previously [6, 27]. For example, a recent prospective study from our team indicated FIB was strongly associated with long-term cardiac and all-cause mortality among CAD patients undergoing PCI, especially when complicated with DM and Pre-DM [7]. Albumin, which is the most abundant protein in human extracellular fluid, plays significant physiological functions as a negative inflammation biomarker, an inhibitor of platelet activation and aggregation, and a mediator of platelet-induced CAD [9, 17]. Many observational studies and meta-analysis have reported a negative correlation between serum albumin levels and cardiovascular outcomes [11, 28, 29]. Considering both plasma FIB and albumin are useful inflammatory biomarkers and strongly associated with cardiovascular events, additional studies are warranted to further evaluate whether their reciprocal relationship, like FAR, could be helpful to identify high-risk individuals in CAD population undergoing PCI, such as diabetic patients.

The fibrinogen-to-albumin ratio, comprising the two easily detective biomarkers mentioned above, is a well-known indicator for assessing the prognosis of patients with some cancers [12–14]. As a promising serum biomarker, FAR has been proved to have better sensitivity and specificity in predicting MACE than FIB and albumin alone [30, 31]. To date, several publications have been conducted on the prognostic value of FAR in different clinical settings [15, 16, 30]. For example, Li et.al found that the value of FAR was associated independently with the severity of CAD and long-term prognosis, which might contribute to improve risk stratification in non-ST-segment elevation myocardial infarction (NSTEMI) patients initially implanted with drug-eluting stents [16]. Recently, Karahan and co-workers reported that FAR was significantly associated with SYNTAX score in predicting the severity of CAD in patients with STEMI [30]. As described above, previous studies have demonstrated that the value of FAR at admission was associated with the severity of CAD and major cardiovascular adverse outcomes in CAD patients after PCI [15, 16, 30]. What’s more, considerable evidence has demonstrated that chronic inflammation is a recognized pathological mechanism of both DM and CAD [2, 18]. It has been previously reported that DM independently increased the risk of adverse events in CAD patients [21]. Our study showed that FAR was positively associated with glycemic metabolism indices (HbA1c and FBG) in our PCI cohort. It is important to investigate the joint effect of FAR and DM on long-term MACCE in CAD patients undergoing PCI. However, there is insufficient literature on the relationship between FAR, different glycemic metabolism and cardiovascular events.

To the best of our knowledge, our study demonstrated that higher level of FAR combined with impaired glycemic metabolism was strongly related to increased risks of long-term MACCEs, for the first time. Furthermore, non-diabetic patients with lower level of FAR also had lower all-cause mortality and cardiac mortality risk than those in the FAR-H/DM group. Multivariate Cox analysis also indicated the highest risk of MACCEs in patients with FAR-H and DM than others (P for trend = 0.005). Interestingly, we observed there were no significant differences among groups in the risk of other components of MACCE, which deserves further exploration in future studies. Given the clinical burden that both diabetes and inflammation exert on cardiovascular risk complications, the joint evaluations of diabetes and FAR might be of clinical significance for management of high-risk individuals in the FAR-H/DM group. However, till now, no treatment can specifically control the level of FAR on a long-term basis [32]. Further studies are warranted to evaluate whether CAD patients undergoing PCI, especially combined with DM, could benefit from medication targeting FAR in the future.

In this study, subgroup analysis was conducted to further explore some common factors involved and to identify in which subsets patients with FAR-H/DM is more meaningful. We found that patients with FAR-L/DM, FAR-H/non-DM and FAR-L/non-DM showed significantly consistency in certain subsets (male and non-CKD). Moreover, it may allow researchers to conduct clinical trials for certain therapies (e.g. colchicine, IL-6 inhibition, or low dose rivaroxaban 2.5 mg) for these subgroups to better guide subsequent treatment towards FAR-H/DM individuals. For instance, the CADENCE trial (NCT04181996) was conducted to determine if the drug colchicine had an effect on plaque inflammation in patients at high risk for events (patients with DM or Pre-DM and recent MI, stroke or transient ischemic attacks). The results focusing on patients with recent MI in this RCT have confirmed the safety and efficacy of colchicine [33]. Combined with the findings of our research, we are looking forward to the favorable results from the diabetic subgroup in the CADENCE trial. The ZEUS trial (NCT05021835) was designed to address whether IL-6 inhibition would reduce cardiovascular events in patients with both CKD and residual inflammatory risk [34]. The ongoing TRACK trial (NCT03969953) will hopefully answer whether low dose rivaroxaban therapy could bring favorable benefit in patients with both CAD and CKD. However, according to subgroup analysis in this study, it seems that in patients without CKD, controlling inflammation and treating diabetes also remain significant, which deserves our attention. Nevertheless, any recommendations made here are tentative, due to the limitations about the observational research.

Another issue to be discussed is the potential mechanisms underlying the association of FAR and DM with unfavorable prognosis. Firstly, previous studies confirmed that FIB could upregulate the expression of proinflammatory cytokines, like interleukin-1 and tumor necrosis factor-α, induce vascular inflammation and endothelial dysfunction, facilitate monocyte or macrophage adhesion, stimulate the proliferation and migration of vascular smooth muscle cells and eventually lead to the formation and vulnerability of atherosclerotic plaque [7, 35]. In addition, evidence from previous literatures suggested that higher concentrations of plasma FIB might contribute to the increase of blood viscosity and peripheral resistance, thereby increasing the risk of thrombosis and ischemic events during follow-up [28, 36]. Secondly, some basic researches proved that physiological concentration of serum albumin could inhibit the expression of vascular cell adhesion molecule-1, increase the elimination of oxygen-free radicals and finally reduce the inflammatory response, suggesting albumin was a protective anti-inflammatory property [17, 37]. Thirdly, inflammation is the shared antecedent of diabetes and atherosclerosis [2, 18]. In line with the previous studies, FAR was positively associated with glycemic metabolism indices and the potential association between DM and FAR had been discussed above [38, 39]. Therefore, further studies are required to elucidate the potential mechanisms. Meanwhile, the importance of routine screening for both novel inflammation biomarkers and impaired glycemic metabolism indices could not be neglected.

This study has several limitations. Firstly, the level of FAR was only calculated at baseline. Dynamic changes in this novel biomarker during follow-up are missing. Secondly, as the nature of the observational studies, potential confounders could not be adequately adjusted. Further randomized clinical trials are necessary to confirm our findings. Thirdly, this study was conducted in Chinese patients with CAD undergoing PCI and whether the findings could be generalized to other populations remains unknown. Fourthly, there was a degree of selection bias because of the strict exclusion criteria that excluded most of the individuals mainly due to FIB data unavailable.

## Conclusion

In this real-world cohort study, higher level of FAR combined with DM was associated with worse 5-year outcomes among patients with CAD undergoing PCI. The level of FAR may help to identify high-risk individuals in this specific population, where more precise risk assessment should be performed.

## Supplementary Information

**Additional file 1: Table S1**. Comparison of baseline characteristics and crude outcomes of participants and non-participants due to exclusion criteria. **Table S2.** Univariate Cox proportional hazard analysis for primary endpoint. **Table S3.** Correlation analysis between glycemic metabolism and FAR in patients with DM, without DM and whole. **Table S4.** Subgroup analysis for the primary endpoint as the unadjusted model. **Table S5.** Subgroup analysis for the primary endpoint as the adjusted model. **Fig. S1.** Restricted cubic splines of FAR levels in relation to crude HR(A) and adjusted HR(B) for the risk of MACCE. Model adjusted for age, sex, BMI, hypertension, previous MI, previous PCI, previous stroke, eGFR, LVEF, LM/three-vessel disease, and SYNTAX score. Red line with 95% confidence interval shaded in light red. *HR* hazard ratio, *CI* confidence interval, *FAR* fibrinogen to albumin ratio, *MACCE* major adverse cardiac and cerebrovascular events.

## Data Availability

Due to ethical restrictions related to the consent given by subjects at the time of study commencement, our datasets are available from the corresponding author upon reasonable request after permission of the Institutional Review Board of State Key Laboratory of Cardiovascular Disease, Fuwai Hospital, National Center for Cardiovascular Diseases.
