# Supplementary material for: High fibrinogen-to-albumin ratio with type 2 diabetes mellitus is associated with poor prognosis in patients undergoing percutaneous coronary intervention: 5-year findings from a large cohort
Source: Cardiovasc Diabetol. 2022 Mar 21;21:46. doi: 10.1186/s12933-022-01477-w (PMC8939137; doi:10.1186/s12933-022-01477-w)
Supplement: Supplementary file 1 — Additional file 1: Table S1. Comparison of baseline characteristics and crude outcomes of participants and non-participants due to exclusion criteria. Table S2. Univariate Cox proportional hazard analysis for primary endpoint. Table S3. Correlation analysis between glycemic metabolism and FAR in patients with DM, without DM and whole. Table S4. Subgroup analysis for the primary endpoint as the unadjusted model. Table S5. Subgroup analysis for the primary endpoint as the adjusted model. Fig. S1. Restricted cubic splines of FAR levels in relation to crude HR(A) and adjusted HR(B) for the risk of MACCE. Model adjusted for age, sex, BMI, hypertension, previous MI, previous PCI, previous stroke, eGFR, LVEF, LM/three-vessel disease, and SYNTAX score. Red line with 95% confidence interval shaded in light red. HR hazard ratio, CI confidence interval, FAR fibrinogen to albumin ratio, MACCE major adverse cardiac and cerebrovascular events. [file 12933_2022_1477_MOESM1_ESM.docx]

**Table S1** Comparison of baseline characteristics and crude outcomes of participants and non-participants due to exclusion criteria.

| **Variable** | **Non-participants**  **(n = 5,426)** | **Participants**  **(n = 5,298)** | **P value** |
| --- | --- | --- | --- |
| Baseline characteristics |  |  |  |
| Age, years | 58.35 ± 10.24 | 58.36 ± 10.36 | 0.948 |
| Male, n (%) | 4,153 (76.5) | 4,119 (77.7) | 0.137 |
| BMI, kg/m^2^ | 25.97 ± 3.21 | 25.89 ± 3.15 | 0.193 |
| DM, n (%) | 2,488 (45.9) | 2,305 (43.5) | 0.015 |
| Hypertension, n (%) | 3,520 (64.9) | 3,386 (63.9) | 0.298 |
| Dyslipidemia, n (%) | 3,554 (65.5) | 3,657 (69.0) | < 0.001 |
| Smoking history, n (%) | 3,163 (58.3) | 3,107 (58.6) | 0.712 |
| Family history of CAD, n (%) | 1,429 (26.4) | 1,222 (23.1) | < 0.001 |
| Previous MI, n (%) | 1,025 (18.9) | 1,036 (19.6) | 0.383 |
| Previous PCI, n (%) | 1,411 (26.0) | 1,229 (23.2) | 0.001 |
| Previous stroke, n (%) | 594 (10.9) | 556 (10.5) | 0.449 |
| Previous PAD, n (%) | 138 (2.5) | 150 (2.8) | 0.356 |
| Clinical presentation, n (%) |  |  | < 0.001 |
| CCS | 2,035 (37.5) | 2,258 (42.6) |  |
| ACS | 3,391 (62.5) | 3,040 (57.4) |  |
| Laboratory tests |  |  |  |
| FIB, g/L | 3.48 ± 0.89 | 3.38 ± 0.83 | 0.017 |
| Albumin, g/L | 43.35 ± 4.27 | 42.40 ± 3.83 | < 0.001 |
| FBG, mmol/L | 6.33 ± 2.18 | 6.02 ± 1.97 | < 0.001 |
| HbA1c, % | 6.64 ± 1.27 | 6.59 ± 1.21 | 0.004 |
| TG, mmol/L | 1.78 ± 1.09 | 1.80 ± 1.09 | 0.587 |
| TC, mmol/L | 4.24 ± 1.07 | 4.17 ± 1.09 | 0.239 |
| HDL-C, mmol/L | 1.04 ± 0.29 | 1.02 ± 0.27 | 0.041 |
| LDL-C, mmol/L | 2.53 ± 0.91 | 2.48 ± 0.91 | 0.316 |
| hs-CRP, mg/L | 3.41 ± 3.97 | 3.07 ± 3.68 | < 0.001 |
| Creatinine, μmol/L | 75.99 ± 16.00 | 75.33 ± 15.95 | 0.801 |
| eGFR, mL/min/1.73 m^2^ | 90.94 ± 15.17 | 91.63 ± 15.10 | 0.795 |
| LVEF, % | 62.28 ± 7.64 | 63.23 ± 7.04 | < 0.001 |
| Medications at admission |  |  |  |
| Aspirin, n (%) | 5,340 (98.4) | 5,245 (99.0) | 0.007 |
| Clopidogrel, n (%) | 5,415 (99.8) | 5,286 (99.8) | 0.790 |
| β-blocker, n (%) | 4,844 (89.3) | 4,829 (91.1) | 0.001 |
| CCB, n (%) | 2,731 (50.3) | 2,485 (46.9) | < 0.001 |
| Statins, n (%) | 5,173 (95.3) | 5,112 (96.5) | 0.003 |
| Nitrate, n (%) | 5,321 (98.1) | 5,162 (97.4) | 0.027 |
| Insulin, n (%) | 632 (25.4) | 613 (11.6) | 0.402 |
| Coronary procedural information |  |  |  |
| LM / three-vessel disease, n (%) | 2,386 (44.0) | 2,382 (45.0) | 0.304 |
| Chronic total occlusion, n (%) | 484 (8.9) | 377 (7.1) | 0.001 |
| Target vessel territory, n (%) |  |  | 0.463 |
| LAD | 2,610 (48.1) | 2,512 (47.4) |  |
| LCX | 961 (17.7) | 955 (18.0) |  |
| RCA | 1,791 (33.0) | 1,757 (33.2) |  |
| Number of stents | 1.60 ± 0.95 | 1.77 ± 0.89 | < 0.001 |
| SYNTAX score | 11.57 ± 8.16 | 11.79 ± 9.06 | 0.163 |
| Complete revascularization, n (%) | 5,061 (93.3) | 5,268 (99.4) | < 0.001 |
| DES implantation, n (%) | 4,900 (90.3) | 5,226 (98.6) | < 0.001 |
| Crude outcomes |  |  |  |
| MACCE | 1,139 (21.0) | 1,099 (20.7) | 0.012 |
| All-cause mortality | 201 (3.7) | 206 (3.9) | 0.454 |
| Non-fatal MI | 261 (4.8) | 310 (5.9) | 0.330 |
| Non-fatal ischemic stroke | 180 (3.3) | 184 (3.5) | 0.579 |
| Unplanned coronary revascularization | 749 (3.8) | 671 (2.7) | < 0.001 |

Values are mean ± SD or n (%). P value for test of difference across the 2 non-participant and eligible participant groups by the chi-square test for categorical variables, student’s t-test for continuous variables or log-rank test

for the risks of crude outcomes.

*BMI* body mass index, *DM* diabetes mellitus, *CAD* coronary artery disease, *MI* myocardial infarction, PCI percutaneous coronary intervention, PAD peripheral artery disease, *CCS* chronic coronary syndrome, *ACS* acute coronary syndrome, *FIB* fibrinogen, *FBG* fasting blood glucose, *HbA1c* glycosylated hemoglobin A1c, *TG* triglyceride, *TC* total cholesterol, *HDL-C* high-density lipoprotein cholesterol, *LDL-C* low-density lipoprotein cholesterol, *hs-CRP* high-sensitivity C-reactive protein, *eGFR* estimated glomerular filtration rate, *LVEF* left ventricular ejection fraction, *CCB* calcium channel blocker, *LM* left main artery, *LAD* left anterior descending artery, *LCX* left circumflex artery, *RCA* right coronary artery, *SYNTAX* synergy between PCI with taxus and cardiac surgery, *DES* drug-eluting stent, *MACCE* major adverse cardiac and cerebrovascular events.

**Table S2** Univariate Cox proportional hazard analysis for primary endpoint

| **Variables** | **Univariate analysis** | | | **Multivariate analysis** | | |
| --- | --- | --- | --- | --- | --- | --- |
|  | **HR** | **95% CI** | **P value** | **HR** | **95% CI** | **P value** |
| Category |  |  |  |  |  |  |
| FAR-H / DM | Reference | - | - | Reference | - | - |
| FAR-L / DM | 0.797 | 0.673-0.945 | 0.009 | 0.812 | 0.683-0.966 | 0.019 |
| FAR-H / Non-DM | 0.742 | 0.626-0.879 | 0.001 | 0.783 | 0.657-0.932 | 0.006 |
| FAR-L / Non-DM | 0.689 | 0.587-0.809 | < 0.001 | 0.752 | 0.636-0.890 | 0.001 |
| Age, per 1 year | 1.010 | 1.004-1.016 | 0.001 | 1.003 | 0.995-1.010 | 0.467 |
| Gender, male as reference | 0.896 | 0.774-1.037 | 0.141 | 0.842 | 0.722-0.983 | 0.029 |
| BMI, per 1 kg/m^2^ | 0.990 | 0.972-1.009 | 0.322 | 0.983 | 0.963-1.003 | 0.093 |
| Hypertension | 1.284 | 1.130-1.459 | <0.001 | 1.251 | 1.095-1.431 | 0.001 |
| Dyslipidemia | 1.050 | 0.923-1.194 | 0.458 |  |  |  |
| Smoking history | 1.127 | 0.998-1.272 | 0.054 |  |  |  |
| Family history of CAD | 1.129 | 0.984-1.294 | 0.083 |  |  |  |
| Prior MI | 1.212 | 1.051-1.397 | 0.008 | 1.042 | 0.890-1.220 | 0.611 |
| Prior PCI | 1.380 | 1.211-1.572 | <0.001 | 1.283 | 1.113-1.478 | 0.001 |
| Prior stroke | 1.244 | 1.040-1.488 | 0.017 | 1.156 | 0.962-1.390 | 0.123 |
| Prior PAD | 0.961 | 0.669-1.382 | 0.831 |  |  |  |
| Clinical presentation, CCS as reference | 1.051 | 0.932-1.184 | 0.420 |  |  |  |
| TG, per 1 mmol/L | 1.002 | 0.950-1.058 | 0.938 |  |  |  |
| TC, per 1 mmol/L | 0.993 | 0.940-1.049 | 0.808 |  |  |  |
| HDL-C, per 1 mmol/L | 0.857 | 0.687-1.069 | 0.171 |  |  |  |
| LDL-C, per 1 mmol/L | 0.998 | 0.935-1.065 | 0.955 |  |  |  |
| hs-CRP, per 1 mg/L | 1.015 | 0.998-1.033 | 0.082 |  |  |  |
| eGFR, per 1 mL/(min*1.73m^2^) | 0.993 | 0.989-0.997 | <0.001 | 0.998 | 0.993-1.003 | 0.469 |
| LVEF, per 1 % | 0.987 | 0.979-0.996 | 0.002 | 0.993 | 0.984-1.002 | 0.108 |
| Aspirin at admission | 0.983 | 0.543-1.781 | 0.955 |  |  |  |
| Clopidogrel at admission | 0.838 | 0.270-2.603 | 0.760 |  |  |  |
| β-blocker at admission | 1.069 | 0.863-1.323 | 0.543 |  |  |  |
| CCB at admission | 1.119 | 0.994-1.260 | 0.062 |  |  |  |
| Statins at admission | 0.937 | 0.686-1.279 | 0.680 |  |  |  |
| Nitrate at admission | 1.542 | 0.979-2.426 | 0.061 |  |  |  |
| LM / three-vessel disease | 1.420 | 1.261-1.598 | <0.001 | 1.275 | 1.118-1.453 | <0.001 |
| Chronic total occlusion | 1.123 | 0.901-1.399 | 0.303 |  |  |  |
| Number of stents, per 1 stent | 1.037 | 0.971-1.107 | 0.277 |  |  |  |
| SYNTAX score, per 1-point | 1.013 | 1.005-1.020 | 0.001 | 1.005 | 0.997-1.013 | 0.191 |
| Complete revascularization | 0.546 | 0.293-1.017 | 0.057 |  |  |  |
| DES implantation | 0.659 | 0.428-1.015 | 0.058 |  |  |  |

Abbreviations as in Table 1 and Table S1.

**Table S3** Correlation analysis between glycemic metabolism and FAR in patients with DM, without DM and whole

| **Variables** | **Adjusted R^2^** | **Coefficient** | **Standard β** | **P value** |
| --- | --- | --- | --- | --- |
| Whole |  |  |  |  |
| FBG, mmol/L | 0.003 | 0.061 | 0.061 | <0.001 |
| HbA1c, % | 0.019 | 0.139 | 0.139 | <0.001 |
| DM |  |  |  |  |
| FBG, mmol/L | 0.001 | 0.037 | 0.037 | 0.863 |
| HbA1c, % | 0.025 | 0.101 | 0.101 | <0.001 |
| Non-DM |  |  |  |  |
| FBG, mmol/L | <0.001 | 0.003 | 0.003 | 0.079 |
| HbA1c, % | 0.010 | 0.003 | 0.003 | <0.001 |

*FAR* fibrinogen‑to‑albumin ratio, *DM* diabetes mellitus, *FBG* fasting blood glucose.

**Table S4** Subgroup analysis for the primary endpoint as the unadjusted model

| **Subgroups** | **Adjusted HR (95% CI)** | | | | **P for interaction** |
| --- | --- | --- | --- | --- | --- |
|  | **FAR-H / DM** | **FAR-L / DM** | **FAR-H / Non-DM** | **FAR-L / Non-DM** |  |
| Age < 65y | Reference | 0.84 (0.48-1.49) | 1.02 (0.59-1.77) | 0.42 (0.23-0.78) | 0.471 |
| Age ≥ 65y | Reference | 0.53 (0.31-0.89) | 0.56 (0.35-0.90) | 0.37 (0.21-0.65) |  |
| Male | Reference | 0.56 (0.36-0.87) | 0.64 (0.42-0.98) | 0.29 (0.19-0.47) | 0.154 |
| Female | Reference | 0.61 (0.28-1.34) | 0.84 (0.44-1.59) | 0.39 (0.17-0.93) |  |
| BMI < 25 | Reference | 0.42 (0.22-0.80) | 0.72 (0.44-1.18) | 0.28 (0.15-0.51) | 0.608 |
| BMI ≥ 25 | Reference | 0.69 (0.43-1.11) | 0.64 (0.39-1.06) | 0.33 (0.19-0.58) |  |
| Non-HT | Reference | 0.32 (0.13-0.82) | 1.01 (0.53-1.90) | 0.31 (0.14-0.67) | 0.182 |
| HT | Reference | 0.66 (0.44-1.00) | 0.59 (0.39-0.91) | 0.33 (0.21-0.54) |  |
| Non-CKD | Reference | 0.59 (0.39-0.89) | 0.73 (0.50-1.07) | 0.33 (0.22-0.51) | 0.977 |
| CKD | Reference | 0.91 (0.35-2.36) | 0.83 (0.34-2.06) | 0.70 (0.20-2.42) |  |
| CCS | Reference | 0.59 (0.33-1.03) | 0.72 (0.41-1.28) | 0.31 (0.17-0.58) | 0.451 |
| ACS | Reference | 0.56 (0.34-0.94) | 0.68 (0.44-1.05) | 0.31 (0.18-0.53) |  |

*FAR* fibrinogen‑to‑albumin ratio, *DM* diabetes mellitus, *BMI* body mass index, *HT* hypertension, *CKD* chronic kidney disease, *CCS* chronic coronary syndrome, *ACS* acute coronary syndrome.

**Table S5** Subgroup analysis for the primary endpoint as the adjusted model

| **Subgroups** | **Adjusted HR (95% CI)** | | | | **P for interaction** |
| --- | --- | --- | --- | --- | --- |
|  | **FAR-H / DM** | **FAR-L / DM** | **FAR-H / Non-DM** | **FAR-L / Non-DM** |  |
| Age < 65y | Reference | 0.88 (0.71-1.08) | 0.80 (0.64-0.99) | 0.75 (0.61-0.91) | 0.368 |
| Age ≥ 65y | Reference | 0.67 (0.49-0.92) | 0.76 (0.57-1.01) | 0.80 (0.58-1.08) |  |
| Male | Reference | 0.78 (0.64-0.94) | 0.74 (0.61-0.91) | 0.68 (0.56-0.82) | 0.118 |
| Female | Reference | 0.87 (0.59-1.28) | 0.89 (0.62-1.27) | 1.14 (0.79-1.64) |  |
| BMI < 25 | Reference | 0.73 (0.55-0.97) | 0.81 (0.62-1.06) | 0.77 (0.60-0.99) | 0.567 |
| BMI ≥ 25 | Reference | 0.85 (0.68-1.06) | 0.76 (0.60-0.96) | 0.73 (0.59-0.92) |  |
| Non-HT | Reference | 0.80 (0.57-1.10) | 0.83 (0.61-1.14) | 0.63 (0.46-0.85) | 0.209 |
| HT | Reference | 0.82 (0.67-1.00) | 0.75 (0.61-0.93) | 0.82 (0.67-1.00) |  |
| Non-CKD | Reference | 0.81 (0.67-0.97) | 0.79 (0.66-0.95) | 0.75 (0.63-0.89) | 0.911 |
| CKD | Reference | 0.99 (0.50-1.98) | 0.70 (0.36-1.35) | 0.85 (0.37-1.93) |  |
| CCS | Reference | 0.80 (0.62-1.06) | 0.88 (0.66-1.17) | 0.76 (0.58-0.98) | 0.512 |
| ACS | Reference | 0.83 (0.66-1.04) | 0.73 (0.58-0.90) | 0.76 (0.61-0.95) |  |

Model adjusted for age, sex, BMI, hypertension, previous MI, previous PCI, previous stroke, eGFR, LVEF, LM / three-vessel disease, and SYNTAX score. In each target analysis, covariates except for the target variable were adjusted in the specific subgroups stratified by Age, sex, BMI or HT.

Abbreviations as in Table S4.


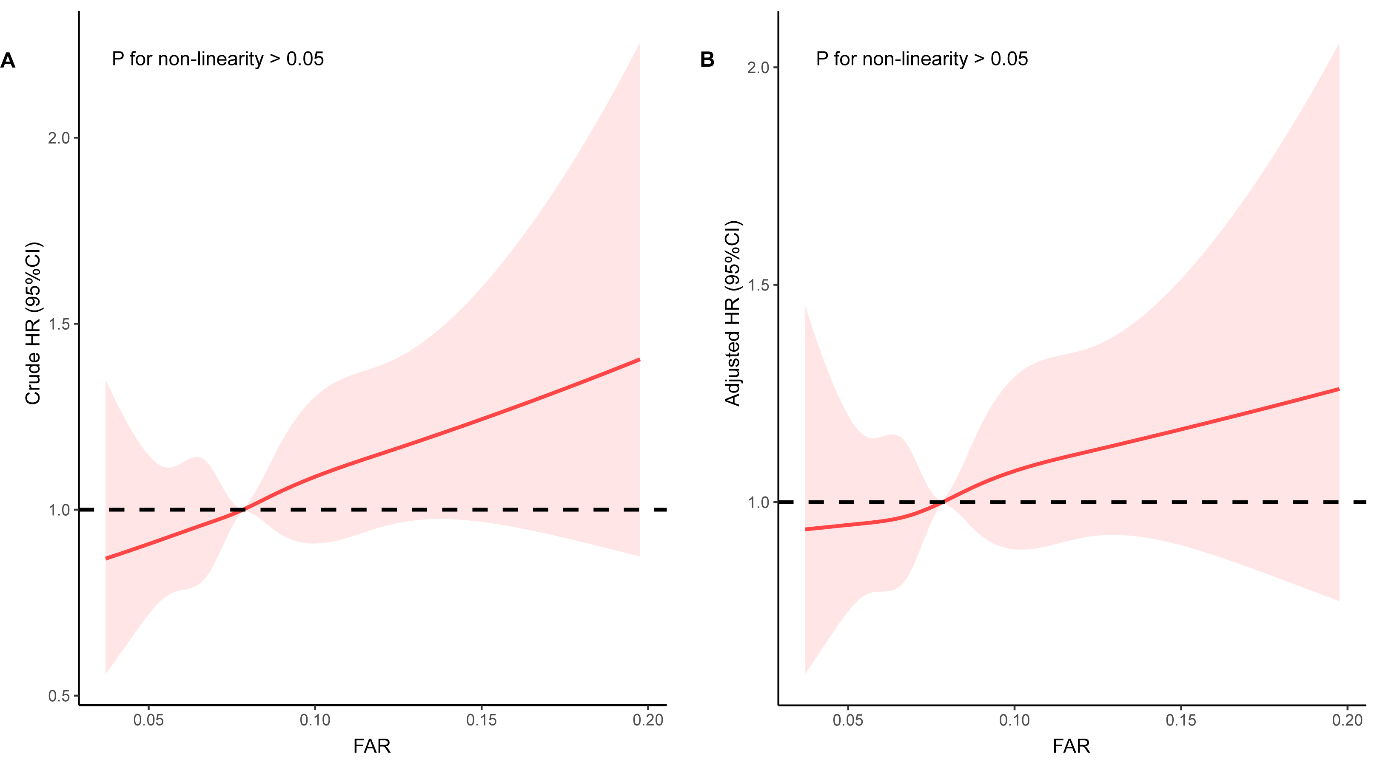


**Fig. S1** Restricted cubic splines of FAR levels in relation to crude HR(A) and adjusted HR(B) for the risk of MACCE. Model adjusted for age, sex, BMI, hypertension, previous MI, previous PCI, previous stroke, eGFR, LVEF, LM / three-vessel disease, and SYNTAX score. Red line with 95% confidence interval shaded in light red. *HR* hazard ratio, *CI* confidence interval, *FAR* fibrinogen to albumin ratio, *MACCE* major adverse cardiac and cerebrovascular events.
